# Differential effects of age, sex and dexamethasone therapy on ACE2/TMPRSS2 expression and susceptibility to SARS-CoV-2 infection

**DOI:** 10.3389/fimmu.2022.1021928

**Published:** 2022-11-03

**Authors:** Shima Shahbaz, Olaide Oyegbami, Suguru Saito, Mohammed Osman, Wendy Sligl, Shokrollah Elahi

**Affiliations:** ^1^ Department of Dentistry, Division of Foundational Sciences, Edmonton, AB, Canada; ^2^ Department of Medicine, Division of Rheumatology, University of Alberta, Edmonton, AB, Canada; ^3^ Department of Critical Care Medicine, University of Alberta, Edmonton, AB, Canada; ^4^ Department of Medicine, Division of Infectious Diseases, University of Alberta, Edmonton, AB, Canada; ^5^ Department of Medical Microbiology and Immunology, Li Ka Shing Institute of Virology, Edmonton, AB, Canada; ^6^ Women and Children Health Research Institute, Faculty of Medicine and Dentistry, University of Alberta, Edmonton, AB, Canada

**Keywords:** COVID-19, ACE2, TMPRSS2, dexamethasone, age, sex

## Abstract

ACE2 and TMPRSS2 are crucial for SARS-CoV-2 entry into the cell. Although ACE2 facilitates viral entry, its loss leads to promoting the devastating clinical symptoms of COVID-19 disease. Thus, enhanced ACE2/TMPRSS2 expression is likely to increase predisposition of target cells to SARS-CoV-2 infection. However, little evidence existed about the biological kinetics of these two enzymes and whether dexamethasone treatment modulates their expression. Here, we show that the expression of ACE2 at the protein and mRNA levels was significantly higher in the lung and heart tissues of neonatal compared to adult mice. However, the expression of TMPRSS2 was developmentally regulated. Our results may introduce a novel concept for the reduced susceptibility of the young to SARS-CoV-2 infection. Moreover, ACE2 expression but not TMPRSS2 was upregulated in adult female lungs compared to their male counterparts. Interestingly, the ACE2 and TMPRSS2 expressions were upregulated by dexamethasone treatment in the lung and heart tissues in both neonatal and adult mice. Furthermore, our findings provide a novel mechanism for the observed differential therapeutic effects of dexamethasone in COVID-19 patients. As such, dexamethasone exhibits different therapeutic effects depending on the disease stage. This was supported by increased ACE2/TMPRSS2 expression and subsequently enhanced infection of normal human bronchial epithelial cells (NHBE) and Vero E6 cells with SARS-CoV-2 once pre-treated with dexamethasone. Therefore, our results suggest that individuals who take dexamethasone for other clinical conditions may become more prone to SARS-CoV-2 infection.

## Introduction

The emergence of the Coronavirus Disease 2019 (COVID-19) has led to a major global pandemic. While severe/critical illness and death are more common in the elderly (1), severe outcomes are uncommon in the young (2, 3). Although infants and children are susceptible to infection, up to 90% of SARS-CoV-2 infected infants/children are asymptomatic or exhibit mild symptoms without a need for hospitalization (4, 5). These observations suggest that there are differences in the immune response to SARS-CoV-2 infection in the young versus the elderly. For instance, it has been proposed that infants respond to micro-organisms through biased immune tolerance rather than resistance strategies – preventing excessive immune response and collateral damage in the young (3). Also, more frequent/recent immunizations in young populations may result in trained immunity and cross-protection against SARS-CoV-2 (5).

Another host factor that may influence the infectivity to SARS-CoV-2 is the differential age-dependent expression of the viral receptor(s) and co-receptor on the target cell (6). Previous studies have indicated that SARS-CoV-2 enters the cell by binding to the Angiotensin-converting enzyme 2 (ACE2) on the cell surface *via* the spike protein (7, 8). Subsequently, the spike protein is cleaved by transmembrane protease serine 2 (TMPRSS2), facilitating target cell entry (7). Thus, SARS-CoV-2 not only gains initial entry *via* ACE2 but also downregulates ACE2, which impairs its protective physiological role (9). The downregulation of ACE2 in the respiratory tract is linked to neutrophil infiltration in response to LPS (10) and may result in angiotensin II accumulation and lung injury in respiratory viral infections (11, 12). Because ACE2 is a carboxypeptidase degrading angiotensin II, B1-bradykinin, and apelin-13, it contributes to cardiovascular physiology (13). Moreover, the enzymatic activity of ACE2 is protective against acute respiratory distress syndrome (ARDS) caused by viral and non-viral lung injury. ACE2 is predominantly expressed in the upper respiratory tract, heart, and other tissues (14, 15).

Therefore, in addition to the respiratory tract, the cardiac tissue could be the target of SARS-CoV-2. For example, it is reported that infected individuals with SARS-CoV-2 could experience cardiovascular disease such as myocarditis (16). A pilot study has reported the detection of SARS-CoV-2 viral RNA in 41% of heart tissues from deceased individuals who died of COVID-19 disease (17).

Although mounting evidence suggests that age and sex may influence ACE2 expression (18), this has been the subject of debate. For example, ACE2 expression in the lung tissue was not different between sexes and age groups in different cohorts of rodents (rats and mice) (19). Similar results were reported for the expression of ACE2 in human lung tissues (19). However, upregulation of ACE2 with age has been seen only in COVID-19 patients requiring mechanical ventilation (20). In contrast, a higher ACE2 expression in the lung of younger compared to older animals, regardless of their sex, has been reported (21). A couple of recent studies reported increased ACE2 gene expression with age in the respiratory tract (22). This discordance illustrates the need for a more unifying explanation as to whether ACE2 and TMPRSS2 are differentially expressed in the young and whether sex influences their expression. Moreover, recent evidence supports a survival advantage with dexamethasone in COVID-19 patients requiring oxygen or invasive mechanical ventilation (23, 24). However, the same treatment has no benefit and may exacerbate disease in patients with milder disease (24).

In the present study, we examined the expression of ACE2 and/or TMPRSS2 in one week old neonatal, 2 and > 6 months old mice at the gene and protein levels. We also examined the effects of dexamethasone treatment on ACE2 and TMPRSS2 expression in lung and heart tissues of both neonatal and adult mice. Finally, as proof of concept, we show that dexamethasone treatment enhances the infectivity of Verso E6 and normal human bronchial epithelial cells (NHBE) to pseudo SARS-CoV-2. Thus, our study may provide a potential and novel explanation for the observed differential effects of dexamethasone in COVID-19 patients.

## Material and methods

### Animal studies

BALB/c mice at different age groups were used for these studies. Similarly, the effects of dexamethasone (Sigma) on ACE2 and TMPRSS2 expression in the lung and heart tissues were assessed 2 days post intraperitoneal (IP) injection using the physiologically relevant concentration of 1 μg/g body weight (25). The research ethics boards at the University of Alberta approved these studies with the protocol # AUP0001021.

### Western blot analyses

Tissues were lysed in lysis buffer supplemented with a protease inhibitor cocktail (Sigma-Aldrich) and protein concentration was determined using a BCA assay kit (Thermo Fisher Scientific). Protein samples were separated by electrophoresis on either 7%, 17%, or 4-15% gradient polyacrylamide gels and then transferred to PVDF membranes. The membranes were blocked with 5% milk and incubated with the anti‐ACE2 (Abcam, ab15348), anti‐TMPRSS2 (Abcam, ab242384), and anti‐β‐actin/GAPDH (Sigma) antibodies using 1:1000 dilution.

Next, membranes were incubated with the appropriate HRP‐conjugated secondary antibodies and developed using an enhanced chemiluminescence detection kit (Thermo Fisher Scientific).

Protein bands of interest were quantified using Image Lab Software v6.0.1 (Bio-Rad).

### Gene expression analysis

RNA was isolated from the lung and heart tissues of mice using RNAeasy mini kit (Qiagen). The concentration of isolated RNA samples was measured using a nanodrop spectrophotometer (ThermoFisher Scientific) and samples with 260/280 ratios between 1.8 and 2.0 were selected for further analysis. RNA (500 ng) was used for cDNA synthesis using the miScript II Reverse Transcription Kit (Qiagen) and the T100 Thermal Cycler (Bio-Rad). The expression of genes was measured by RT-PCR using CFX96 Touch Real-Time PCR Detection System (Bio-Rad). The Quantitect Primer assay (Qiagen) was carried out for the following genes: ACE2, TMPRSS2 with Beta-2-microglobulin used as the reference gene. Data analysis was done using the 2 ^-CT^ method as we reported elsewhere (26, 27).

### Immunofluorescence staining

Tissues were harvested and fixed in 4% PFA overnight. Slides were deparaffinized by washing twice in xylene for 10 min, 2 times in 100% EtOH for 10 min, 1 time in 95% EtOH for 5 min, 1 time in 70% EtOH for 5 min, and finally a 5-min wash in H_2_O. Next, slides were incubated in pre-warmed citrate buffer (pH 6.0) in a water bath at 92°C for 10 min, then removed and allowed to cool to room temperature followed by 3 washes with 1x PBS with 5 min intervals. First, slides were incubated in 10% donkey serum in PBST at RT for 1 hour to minimize non-specific antibody binding. After 3 washes with 1x PBS at 5 min intervals, samples were incubated in 100 µL of ACE2 primary antibody (1:200 Abcam ab15348) overnight in a moist chamber at 4°C. For the staining of TMPRSS2, samples were incubated in 100 µL of TMPRSS2 primary antibody (1:200 Abcam ab92323) overnight in a moist chamber at 4°C. For the negative control, samples were incubated with 100 µL PBS overnight in a moist chamber at 4°C. The next day slides were washed 3 times with 1x PBS at 5 min intervals. Finally, 100 µL of Alexa Flour 488 secondary antibody (1:1000 Invitrogen A32790) was added to samples in the dark and incubated for 1 hour at room temperature, and then washed 3 times with 1x PBS for 5 min each wash. The samples were then incubated with 100 µL of DAPI (1:1000 Invitrogen D1306) for 10 min. Slides were then washed 3 times with 1x PBS at 5 min intervals. A drop of ProLong Mountant (Invitrogen P36934) was added to each sample before coverslips were placed.

### Infection assay with the pseudo SARS-CoV-2

Vero E6 cells were seeded at 30,000 cells/well in complete RPMI culture media in the presence or absence of dexamethasone (0.5 μg/ml and 1 μg/ml, Sigma) in a 48 well flat-bottomed plate for 24 hr. Similarly, NHBE cells originally purchased from the ATCC but no information was provided for the age/sex of the donor. These cells were seeded at 30,000 cells/well in bronchia epithelial cell growth media (BEGM, Sigma) in the presence or absence of dexamethasone (1 μg/ml) in a 48 well flat-bottomed plate for 24 hr. Then, cells were washed with warm PBS and exposed to the pseudo SARS-CoV-2 spike Delta variant green reporter (Montana Molecular, MT, USA) according to the manufacturers’ instruction overnight. Then cells were trypsinized and extensively washed of any extracellular pseudo virus before analysis by imaging and/or flow cytometry.

### Flow cytometry

The expression of ACE2/TMPRSS2 was analyzed by using the anti-ACE2 (535919) from R&D, and the anti-TMPRSS2 (EPR3862) from abcam according to our methods (28–30). Besides, Live/dead fixable dead cell stain (ThermoFisher) was used to exclude dead cells in flow cytometry. Paraformaldehyde fixed cells were acquired by flow cytometry using a LSRFORTESSA flow cytometer (BD) and analyzed with FlowJo software (version 10).

### Statistical analysis

Statistical analysis was performed using the Prism software. Based on the distribution of data the appropriate test was used. The non-parametric tests such as the Mann-Whitney U-test or Kruskal–Wallis one-way analysis of variance was used. Also, Wilcoxon matched-pairs signed rank test was used for paired studies (e.g. *in vitro* treatment). The *P*-values are shown in the graphs and measures are expressed as mean ± SEM and *P-*value < 0.05 was considered to be statistically significant.

## Results

### Higher ACE2 but lower TMPRSS2 in lung tissues of neonatal compared to adult mice

**Figure 1:**
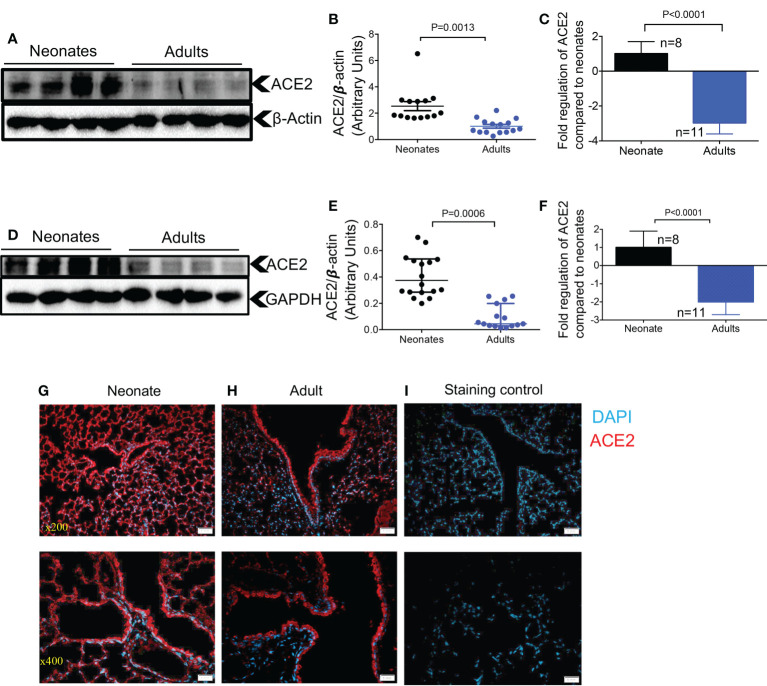
Differential expression of ACE2 and TMPRSS2 in the lung and heart tissues of neonatal compared to adult mice. **(A)** Representative western immunoblots, and **(B)** cumulative data of ACE2 protein in the lung tissues of neonatal (1-week-old) versus adult mice (> 6 months). **(C)** Cumulative data showing fold regulation of ACE2 mRNA in the lung tissues of neonatal versus adult mice (> 6 months). **(D)** Representative western immunoblots, and **(E)** cumulative data of ACE2 in the heart tissues of neonatal versus adult mice (> 6 months). **(F)** Cumulative data showing fold regulation of ACE2 mRNA in the heart tissues of neonatal versus adult mice (> 6 months). **(G)** Representative immunofluorescence (IF) images of ACE2 expression in the lung tissue of a neonatal mouse, and **(H)** an adult mouse (5 sections/tissue, n= >3/group). **(I)** IF image of the lung section stained with the secondary antibody as control. Scale bar: 100 μm. Magnification x200 and x400. Sample size (n) is shown for each group. All blots were repeated for reproducibility and lanes were loaded with equal amounts of protein. Quantification of ACE2 (97 KDa) and TMPRSS2 (54 KDa) normalised to the loading control. Error bars indicate mean ± SEM from more than two independent experiments. Each dot represents data from an animal.

To compare the expression levels of ACE2 in the lung of neonatal compared to older mice, we measured total ACE2 protein levels by western blot in lung tissues. We found that neonatal mice (one week old) had significantly higher ACE2 protein levels compared to older adult mice (> 6 months) in bulk lung tissues (**Figures 1A, B**). A similar pattern was noted at the gene expression level when ACE2 mRNA was quantified in lung tissues of neonatal versus older adult mice (**Figure 1C**). This was also the case for ACE2 expression at the protein and gene levels in heart tissues of neonatal compared to older adult mice (**Figures 1D-F**). It is worth mentioning that although ACE2 is observed as a single band in most tissues of mice, it is usually observed as double bands in lung tissue as reported elsewhere (29). We further investigated the expression of ACE2 in the lung tissue by immunofluorescence staining (IF). Although we did not quantify the expression level, these observations suggest a more intense and less scattered ACE2 expression in the lung tissue of neonatal versus adult mice (**Figures 1G-I**). However, our observations did not support a differential expression level for ACE2 in neonatal female/male mice.

**Figure 2:**
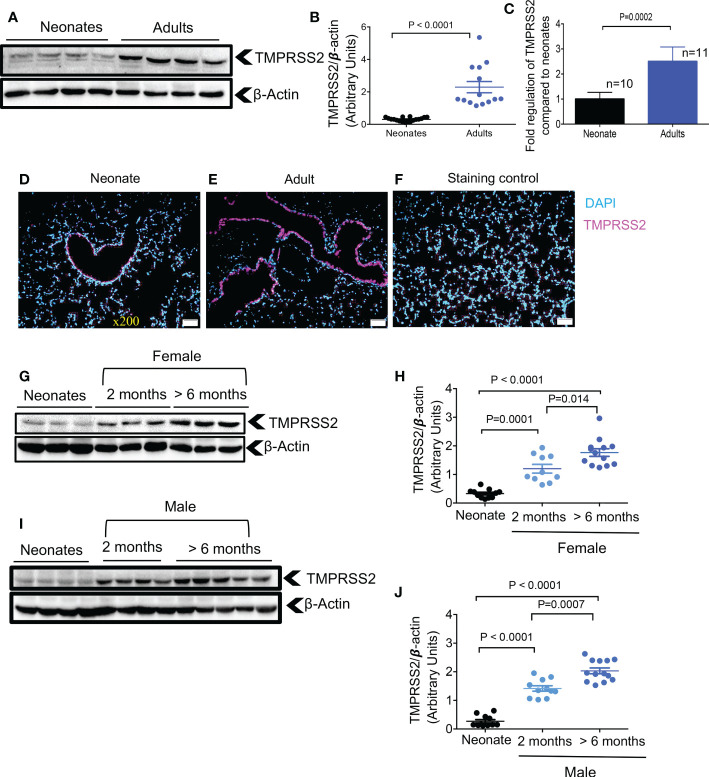
Developmental regulation of TMPRSS2 in the lung tissues of mice. **(A)** Representative western immunoblots, and **(B)** cumulative data of TMPRSS2 protein in the lung tissues of neonatal versus adult mice (> 6 months). **(C)** Cumulative data showing fold regulation of TMPRSS2 mRNA in the lung tissues of neonatal versus adult mice (> 6 months). **(D)** Representative immunofluorescence (IF) images of TMPRSS2 expression in the lung tissue of a neonatal mouse, and **(E)** an adult mouse (5 sections/tissue, n= >3/group). **(F)** IF image of the lung section stained with the secondary antibody as control. Scale bar: 100 μm. Magnification x200. **(G)** Representative western immunoblots, and **(H)** cumulative data of TMPRSS2 protein in the lung tissues of neonatal (8 days old), young adults (2 months) versus older adult (> 6 months) female mice. **(I)** Representative western immunoblots, and **(J)** cumulative data of TMPRSS2 protein in the lung tissues of neonatal, young adults (2 months) versus older adult (> 6 months) male mice. All blots were repeated for reproducibility and lanes were loaded with equal amounts of protein. Quantification of ACE2 and TMPRSS2 normalised to the loading control. Error bars indicate mean ± SEM from at least three independent experiments.

In a recent study, the developmental regulation of TMPRSS2 expression in the human lung epithelium was suggested to be the primary determinant of age-related differences in SARS-CoV-2 infection susceptibility (6). To determine whether this was the case in mice, we examined TMPRSS2 expression by western blot and qPCR in the lung tissues of neonatal versus older adult mice. We found that older adult animals, had significantly higher TMPRSS2 expression compared to neonates at the protein and gene levels in their lung tissues (**Figures 2A-C**). These observations were further confirmed by the IF staining that showed scarce expression of TMPRSS2 in the lung of neonatal versus older adult mice (**Figures 2D-F**). We further observed that TMPRSS2 expression was developmentally regulated in the lung tissues regardless of the animal sex as noted in female (**Figures 2G, H**) and male mice (**Figures 2I, J**). However, we were unable to detect TMPRSS2 expression in heart tissue as reported elsewhere (31). These results confirm the developmental regulation of TMPRSS2 in lung tissues of mice.

### Higher expression of ACE2 but not TMPRSS2 in the lung tissue of female mice

**Figure 3:**
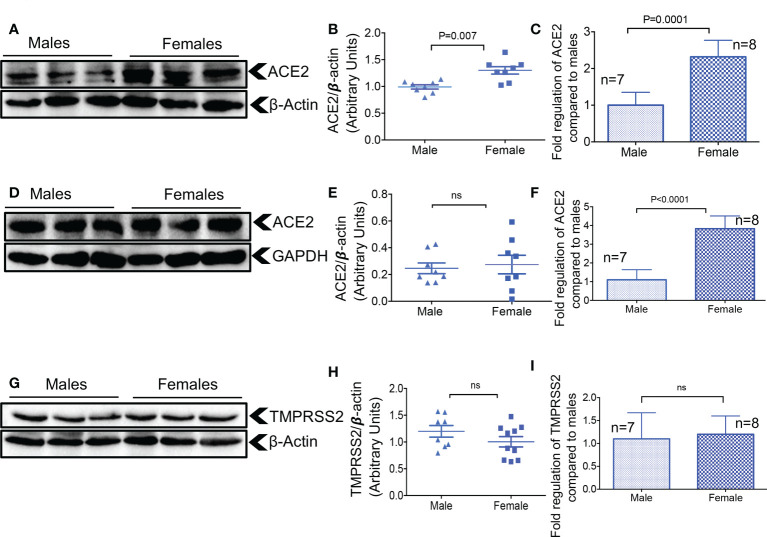
Elevated ACE2 at the gene and protein levels in the lungs of female mice. **(A)** Representative western immunoblots, and **(B)** cumulative data of ACE2 protein in the lung tissues of adult male and female mice. **(C)** Cumulative data showing fold regulation of ACE2 mRNA in the lung tissues of male versus female adult mice. **(D)** Representative western immunoblots, and **(E)** cumulative data of ACE2 protein in the heart tissues of adult male and female mice. **(F)** Cumulative data showing fold regulation of ACE2 mRNA in the heart tissues of male versus female adult mice. **(G)** Representative western immunoblots, and **(H)** cumulative data of TMPRSS2 protein in the lung tissues of male versus female older adult mice. **(I)** Cumulative data showing fold regulation of TMPRSS2 mRNA in the lung tissues of male versus female adult mice. All blots were repeated for reproducibility and lanes were loaded with equal amounts of protein. Quantification of ACE2 and TMPRSS2 normalised to the loading control. Error bars indicate mean ± SEM from at least two independent experiments. ns, Not significant.

Hormonal and genetic factors are reported to result in ACE2 upregulation in females (32), which may, in part, explain differences in COVID-19 outcomes in females versus males. Thus, we analyzed the expression of ACE2 and TMPRSS2 in the lung tissues of older (> 6 months old) female versus male mice. We found that females had significantly higher ACE2 expression at the protein and gene levels in lung tissue compared to males (**Figures 3A-C**). However, this difference was noted only at the gene level but not at the protein level in the heart tissues of females (**Figures 3D-F**). When TMPRSS2 expression was analyzed, we did not find any significant differences between males and females at the gene and protein levels in lung tissues (**Figures 3G-I**). In agreement with another report (31), we were unable to detect TMPRSS2 gene expression in heart tissue. These results highlight the differential effects of sex on ACE2 and TMPRSS2 expression in older mice.

### Dexamethasone upregulates the expression of ACE2 and TMPRSS2 in neonatal mice

**Figure 4:**
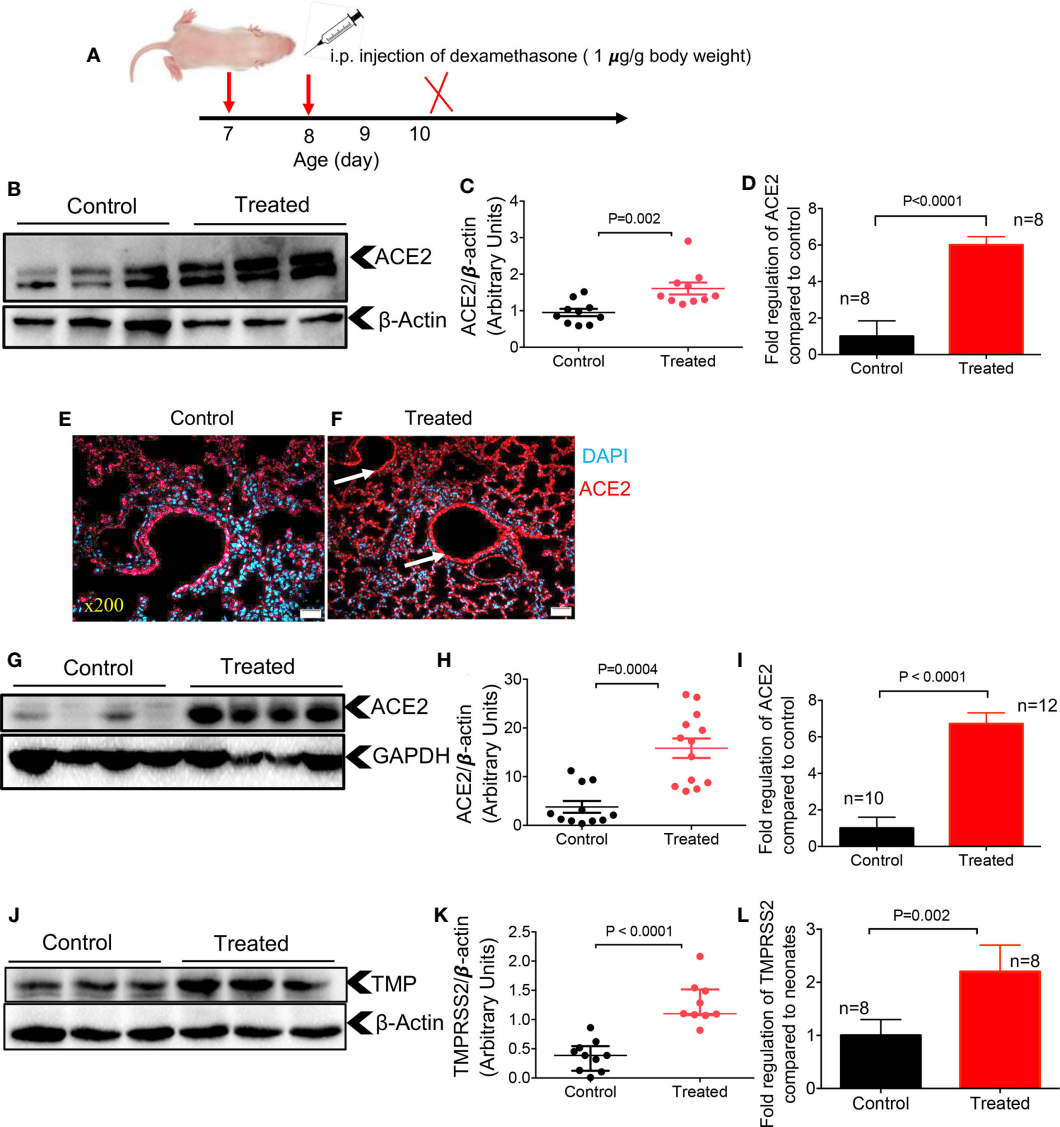
Dexamethasone upregulates the expression of ACE2 and TMPRSS2 in the tissues of neonatal mice. **(A)** Dexamethasone treatment strategy. **(B)** Representative western immunoblots, and **(C)** cumulative data of ACE2 protein in the lung tissues of neonatal mice treated with dexamethasone (IP, 1 μg/g body weight for two consecutive days) versus those treated with PBS (control). **(D)** Cumulative data showing fold regulation of ACE2 mRNA in the lung tissues of treated neonates versus control. **(E)** Representative immunofluorescence (IF) images of ACE2 expression in the lung tissue of a control versus **(F)** dexamethasone treated neonatal mouse (5 sections/tissue, n= >3/group). Scale bar: 100 μm. Magnification x200. **(G)** Representative western immunoblots, and **(H)** cumulative data of ACE2 protein in the heart tissues of control versus dexamethasone treated neonatal mice **(I)** Cumulative data showing fold regulation of ACE2 mRNA in the heart tissues of neonatal mice treated with dexamethasone compared to controls. **(J)** Representative western immunoblots, and **(K)** cumulative data of TMPRSS2 (TMP) protein in the lung tissues of control versus dexamethasone treated neonatal mice **(L)** Cumulative data showing fold regulation of TMPRSS2 mRNA in the lung tissues of neonatal mice treated with dexamethasone compared to controls. All blots were repeated for reproducibility and lanes were loaded with equal amounts of protein. Quantification of ACE2 and TMPRSS2 normalised to the loading control. Error bars indicate mean ± SEM from more than three independent experiments.

Treatment with dexamethasone in hospitalized COVID patients has been shown to reduce mortality among those who require oxygen and/or mechanical ventilation (23). However, there is no evidence, to our knowledge, that dexamethasone may impact the expression of ACE2 and TMPRSS2 in these patients. Therefore, we aimed to determine the effects of dexamethasone on the expression of ACE2 and TMPRSS2 in neonatal mice. One-week old mice were administered intraperitoneal (IP) dexamethasone (1 μg/g body weight) for two consecutive days and their lung and heart tissues were harvested a day later (**Figure 4A**). Control animals were injected (IP) with phosphate buffered-saline (PBS) because the dexamethasone was water soluble and then was diluted in PBS. We found that dexamethasone treatment resulted in a significant upregulation of ACE2 at the protein and gene levels in the lung tissues of one-week old mice (**Figures 4B-D**). Furthermore, using the IF staining, we noted a more intense and less scattered ACE2 expression in the lung tissues of treated versus control mice (**Figures 4E, F**). However, the IF staining was not quantified and only implies a higher ACE2 expression in the treated group. Moreover, the expression of ACE2 at the protein and gene levels in heart tissues confirmed similar results (**Figures 4G-I**). Next, we assessed the expression of TMPRSS2 in the lungs of neonatal mice after treatment with dexamethasone. These studies yielded results that were consistent with the ACE2 expression (**Figures 4J-L**). In summary, these findings support the role of dexamethasone in upregulating the expression of ACE2 and TMPRSS2 in the lung and ACE2 in the heart tissues of neonatal mice.

### Dexamethasone upregulates the expression of ACE2 and TMPRSS2 in adult mice

**Figure 5:**
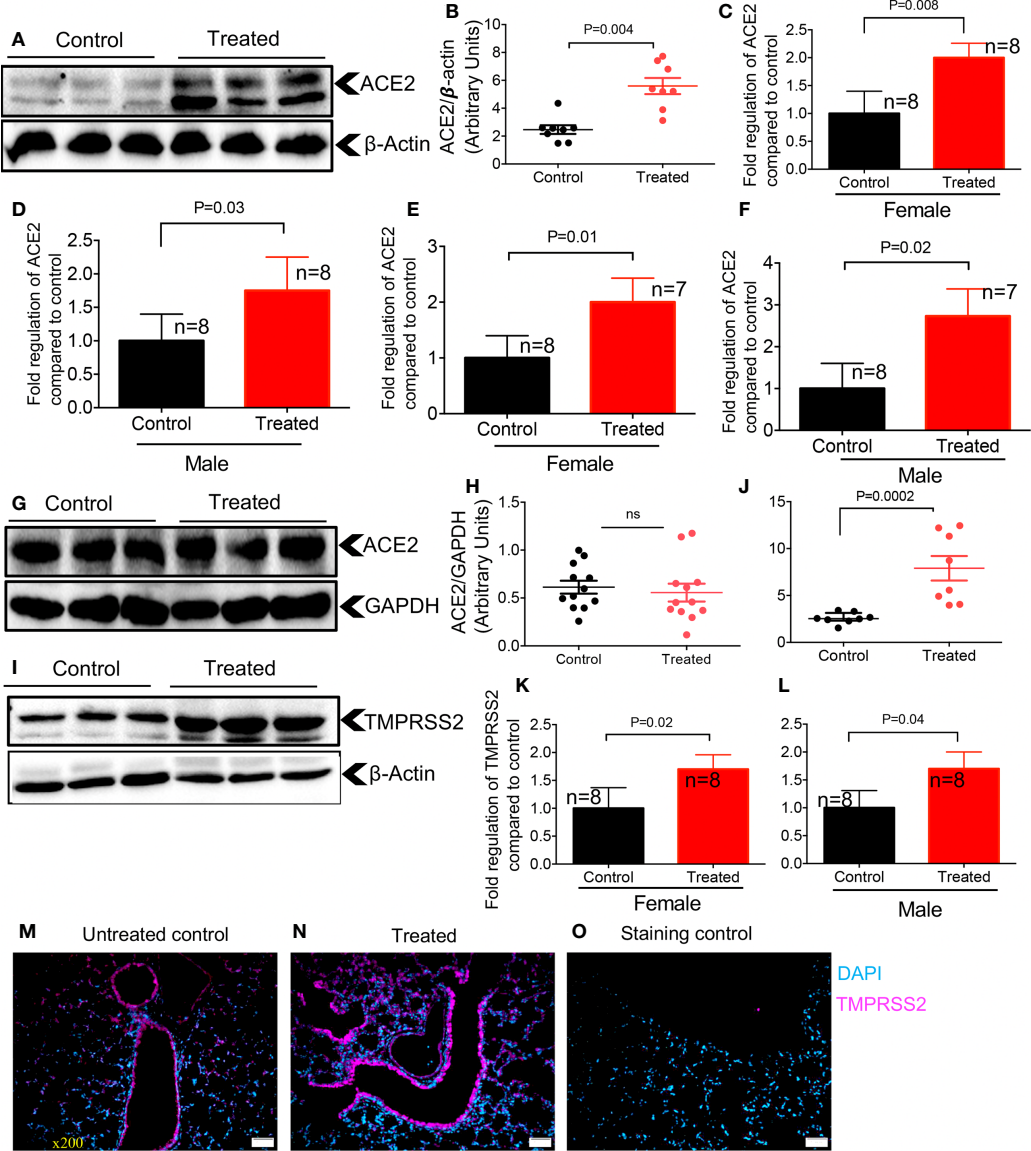
Dexamethasone upregulates the expression of ACE2 and TMPRSS2 in the lung and heart tissues of adult mice. **(A)** Representative western immunoblots, and **(B)** cumulative data of ACE2 protein in the lung tissues of adult mice (> 6 months) treated with dexamethasone (IP, 1 μg/g body weight for two consecutive days) versus those treated with PBS (control). **(C)** Cumulative data showing fold regulation of ACE2 mRNA in the lung tissues of treated male, and **(D)** female versus control mice. **(E)** Cumulative data showing fold regulation of ACE2 mRNA in the heart tissues of treated male, and **(F)** female versus control mice. **(G)** Representative western immunoblots, and **(H)** cumulative data of ACE2 protein in the heart tissues of adult mice (> 6 months) treated with dexamethasone versus controls. **(I)** Representative western immunoblots, and **(J)** cumulative data of TMPRSS2 protein in the lung tissues of adult mice (> 6 months) treated with dexamethasone versus controls. **(K)** Cumulative data showing fold regulation of YMPRSS2 mRNA in the lung tissues of treated male, and **(L)** female versus controls. **(M)** IF images of TMPRSS2 expression in the lung tissue of a control versus **(N)** dexamethasone treated adult mouse. **(O)** Stained with the secondary antibody (5 sections/tissue, n= >3/group). Scale bar: 100 μm. Magnification x200. All blots were repeated for reproducibility and lanes were loaded with equal amounts of protein. Quantification of ACE2 and TMPRSS2 normalised to the loading control. Error bars indicate mean ± SEM from more than three independent experiments. ns, Not significant.

Since our results showed differential expression of ACE2 and TMPRSS2 in the lung and heart tissues of neonatal mice (**Figure 1**), we hypothesized that dexamethasone may also exhibit differential effects on the expression of ACE2 and TMPRSS2 in older adult mice. Strikingly, we found that dexamethasone treatment (1 μg/g body weight, IP) for two consecutive days increased the expression of ACE2 at the protein level in adult mice (> 6 months) regardless of their sex (**Figures 5A, B**). We also observed similar effects in 2-month-old mice. To better understand the role of sex following treatment with dexamethasone, we measured the mRNA level for ACE2 in lung tissues of both male and female adult mice. We observed that dexamethasone treatment upregulated the mRNA expression for ACE2 in lung tissues of both female and male adult mice, respectively (**Figures 5C, D**). Similar observations were made for ACE2 expression at the gene level in the heart tissues of mice once treated with dexamethasone regardless of their sex (**Figures 5E, F**). However, we did not find any changes in ACE2 expression at the protein level in heart tissues of adult mice regardless of their sex despite treating additional animals (**Figures 5G, H**).

Next, we quantified the expression of TMPRSS2 in the lung tissue of animals treated with dexamethasone versus controls, which showed a significantly enhanced TMPRSS2 at the protein level in treated animals (**Figures 5I, J**). We further found that dexamethasone treatment significantly upregulated TMPRSS2 at the mRNA level in treated versus control female and male mice, respectively (**Figures 5K, L**). Finally, the IF staining on sections obtained from the lung tissues of control and treated mice supported the enhanced expression of TMPRSS2 following treatment with dexamethasone (**Figures 5M-O**). Thus, these observations confirmed that dexamethasone enhances ACE2 and TMPRSS2 expression in the lung tissues of adult mice.

### Pre-treatment with dexamethasone enhances susceptibility of NHBE and Vero E6 cells to infection with pseudo SARS-CoV-2

**Figure 6:**
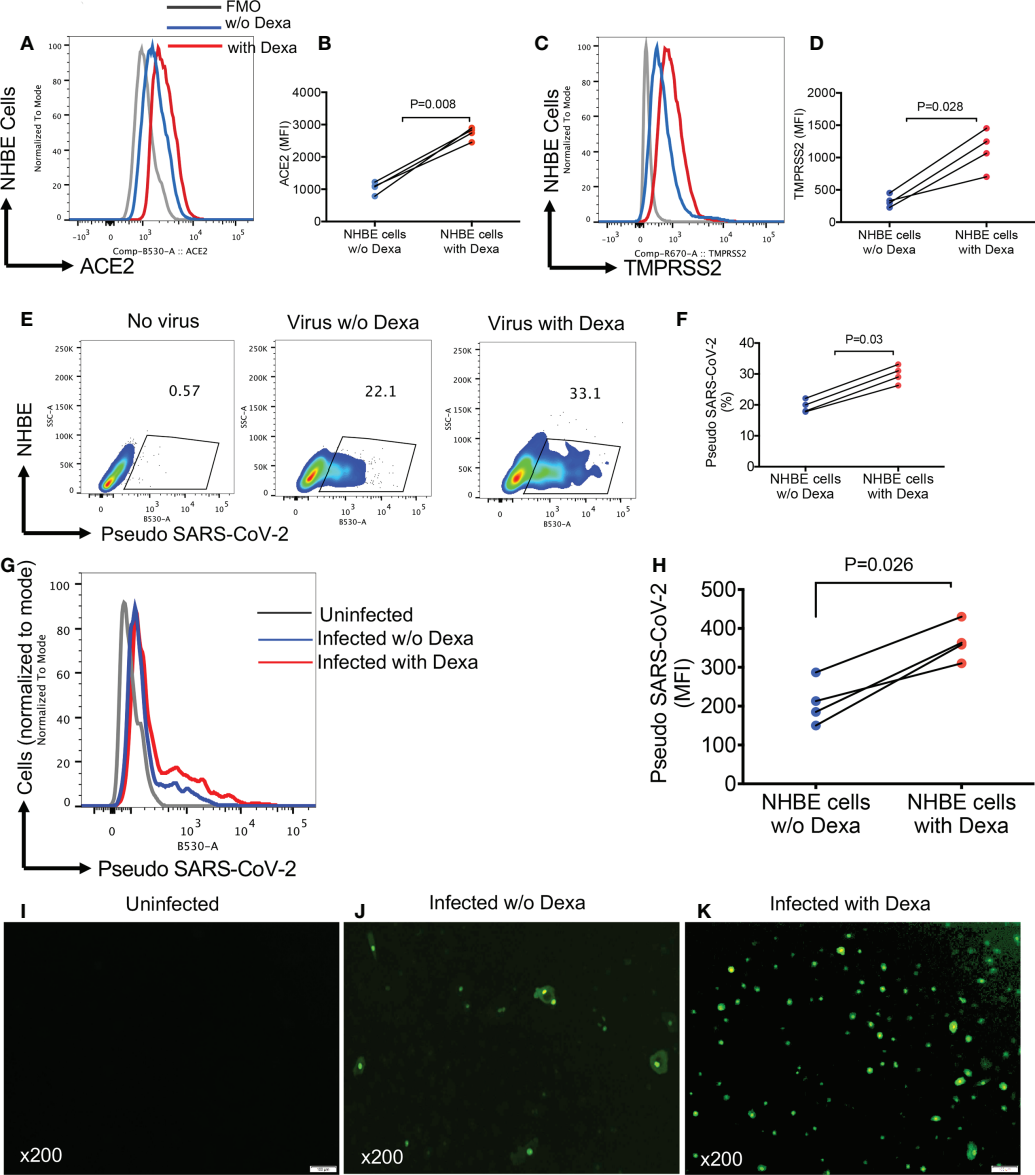
Dexamethasone treatment enhances the infectivity of NHBE cells to Pseudo SARS-CoV-2 infection. **(A)** Representative plots, and **(B)** cumulative data of ACE2 expression as measured by the mean fluorescence intensity (MFI) in NHBE cells following overnight treatment with (0.5 µg/ml) or without dexamethasone treatment. **(C)** Representative plots, and **(D)** cumulative data of TMPRSS2 expression (MFI) in NHBE cells following overnight treatment with (0.5 µg/ml) or without dexamethasone treatment. **(E)** Representative flow cytometry plots, and **(F)** cumulative data showing percentages of NHBE cells untreated or treated with dexamethasone (0.5 μg/ml) and infected with the pseudo SARS-CoV-2. **(G)** Representative flow cytometry histogram plots, and **(H)** cumulative data showing the intensity [the mean fluorescence intensity (MFI)] of pseudo SARS-CoV-2 infection in NHBE cells untreated or treated with dexamethasone (0.5 μg/ml). **(I)** Representative image of uninfected NHBE cells. **(J)** Representative images of dexamethasone untreated but infected NHBE cells with pseudo SARS-CoV-2. **(K)** Representative images of dexamethasone treated (0.5 µg/ml) and infected NHBE cells with pseudo SARS-CoV-2, magnification x200. Error bars indicate mean ± SEM from at least two independent experiments. Each dot represents results from a single experiment. Dexamethasone (Dexa), w/o (without). Fluorescence minus one (FMO).

**Figure 7:**
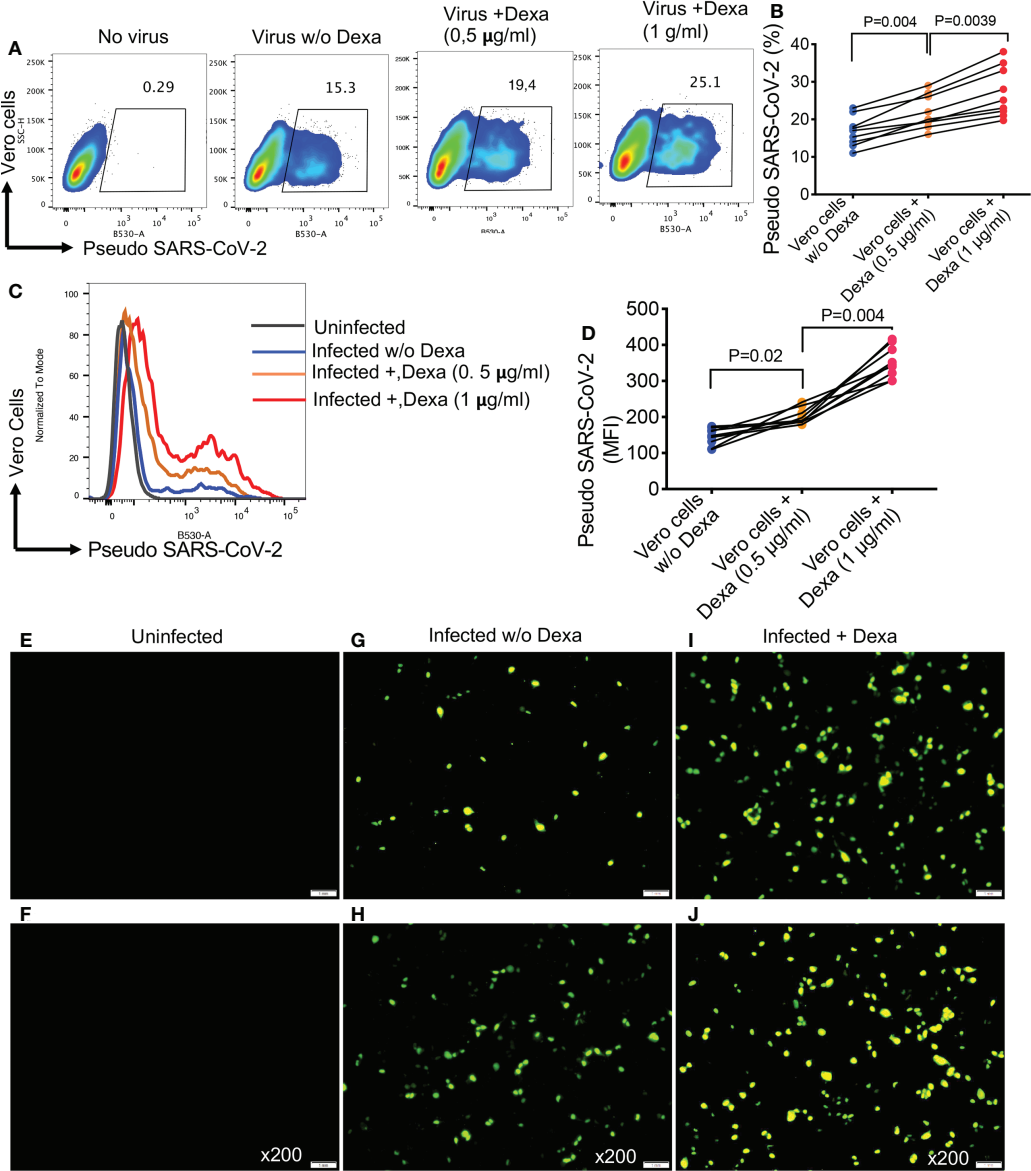
Dexamethasone treatment enhances the infectivity of Vero E6 cells to Pseudo SARS-CoV-2 infection. **(A)** Representative flow cytometry plots, and **(B)** cumulative data showing percentages of Vero E6 cells untreated or treated with dexamethasone (0.5 μg/ml and 1 μg/ml) and infected with the pseudo SARS-CoV-2. **(C)** Representative flow cytometry histogram plots, and **(D)** cumulative data showing the MFI of pseudo SARS-CoV-2 infection in Vero E6 cells untreated or treated with dexamethasone (0.5 μg/ml and 1 μg/ml). **(E, F)** Representative images of uninfected Vero E6 cells. **(G, H)** Representative images of dexamethasone untreated but infected Vero E6 cells with pseudo SARS-CoV-2. **(I, J)** Representative images of dexamethasone treated and infected Vero E6 cells with pseudo SARS-CoV-2, magnification x200. Error bars indicate mean ± SEM from at least two independent experiments. Each dot represents results from a single experiment. Dexamethasone (Dexa), Vero E6 cells (Vero cells), virus (pseudo SARS-CoV-2).

Considering the upregulation of ACE2 and TMPRSS2 in the lung tissues of mice following treatment with dexamethasone, we decided to recapitulate these observations in NHBE cells, as a proof of concept. We found that overnight treatment with dexamethasone (1 μg/ml), significantly increased the expression of ACE2 and TMPRSS2 in NHBE cells (**Figures 6A-D**). This observation was similar to that seen in animal studies (**Figures 4** and **5**). As anticipated, upregulation of SARS-CoV-2 receptor and co-receptor, not only increased the proportion of infected NHBE cells to pseudo SARS-CoV-2 infection (**Figures 6E, F**) but also significantly enhanced the intensity of infection in these cells (**Figures 6G, H**). This was further confirmed by microscopic examinations (**Figures 6I-K**). Wells without treatment served as controls. In addition, we performed similar studies on Vero E6 cells to reconfirm and validate our observations in another cell line. Vero E6 cells are widely used for SARS-CoV-2 infection assays *in vitro* (33). We observed that overnight treatment of Vero E6 cells with dexamethasone (0.5 and 1 μg/ml) significantly increased their susceptibility to infection in a dose dependent manner when exposed to pseudo SARS-CoV-2 and analyzed by flow cytometer (**Figures 7A, B**). Also, the intensity of infection was more pronounced in dexamethasone treated versus untreated Vero E6 cells in a dose dependent manner (**Figures 7C, D**). Finally, our microscopic examinations further confirmed these observations (**Figures 7E-J**). Therefore, these results imply that dexamethasone increases NHBE and Vero E6 cells infectivity to SARS-CoV-2 *via* the upregulation of ACE2 and TMPRSS2.

## Discussion

Here, we report several notable findings in this basic science study with important clinical implications. First, we find that TMPRSS2 is dynamically and developmentally regulated by age. However, ACE2 is highly expressed at the gene and protein levels in the very young and it is not developmentally regulated in mice. Our results are consistent with an earlier study showing greater expression of ACE2 in the lung of young Rhesus monkeys and mice (21, 34). However, our findings contradict another report showing continued expression of ACE2 in the lung as mice age (35). However, this study only analyzed the expression of ACE2 in the lungs of 2-month-old versus older animals (35). In fact, these findings are consistent with our observations except that this group did not investigate the expression of ACE2 in neonatal mice. Similarly, others have identified increased ACE2 expression by age at the gene and protein levels in human lungs (20). However, these results were based on the expression levels of ACE2 in the lungs of older versus younger adults (20). Our results suggest that differential ACE2 expression may contribute to increased or decreased susceptibility to SARS-CoV-2 infection. The second interesting finding of our study is our observation that ACE2 is highly expressed at the gene and protein levels in the lungs of older female mice compared to their male counterparts. Although ACE2 expression is associated with cellular susceptibility to SARS-CoV-2 infection (7, 29), lower tissue levels are correlated with worse clinical outcomes and lung injury (36).Greater ACE2 expression could enhance viral entry at the cellular level, but may provide protection at the organ/tissue level. It is worth mentioning that despite higher expression of ACE2 in the lung of older female mice, TMPRSS2 expression was unchanged. This suggests that females might not be at a greater risk of infection because of the unchanged expression of the co-receptor in their lungs. Instead, this in part may explain why women are at lower risk of complications and mortality associated with COVID-19 infection (18, 37). An additional observation of our study was changes in ACE2 and TMPRSS2 expression in the heart tissues of neonatal versus older mice. We observed a higher expression of ACE2 in the heart tissues of neonatal that older animals. This may increase the likelihood of infection with SARS-CoV-2, as suggested by others, in the lung (38). Notably, in the present study, we found very low to the undetectable levels of TMPRSS2 in the heart tissues, suggesting the inability of SARS-CoV-2 to enter the myocardium. Our results concur with human studies detecting relatively very low TMPRSS2 expression in myocardium (39, 40). This implies that although ACE2 is highly expressed in the heart, without the presence of a co-receptor (e.g. TMPRSS2), myocardial infectivity of SARS-CoV-2 is low. Emerging data indicate an association between COVID-19 and myocarditis (41, 42). Intriguingly, the association between COVID-19 and myocarditis has been reported to be the highest among those younger than 16 years (43). Although causality is unknown, we suggest that higher expression of ACE2 in the heart of the young could partially explain the observed age-related association.

Another finding was the effects of dexamethasone on ACE2 and TMPRSS2 expression in tissues of neonatal and older mice. Strikingly, our results revealed that dexamethasone treatment enhances the expression of ACE2 and TMPRSS2 at the gene and protein levels in the lung tissues of mice regardless of their age/sex. Based on these observations we speculate that dexamethasone may exhibit the same effects in human subjects resulting in enhanced susceptibility to SARS-CoV-2 infection. This hypothesis was further confirmed when we noted significantly increased ACE2 and TMPRSS2 expression in NHBE cells upon treatment with dexamethasone. This was resulted in enhanced infectivity of NHBE cells with pseudo-SARS-CoV-2 once pre-treated with dexamethasone *in vitro*. However, these are hypotheses generated based on our intriguing animal and *in vitro* results, which require validation in human subjects.

Moreover, viral entry represents just one of several elements involved in SARS-CoV-2 infection, each of which involves different genes and pathways that may potentially be influenced by dexamethasone in the lung/heart and elsewhere. As such, whether dexamethasone modulates other genes and pathways associated with viral replication/infection merits further investigation.

Another important discovery of this study is the very low expression of TMPRSS2 in both lungs and heart tissues of neonatal compared to older mice. Similar to our results, very low expression of TMPRSS2 has been reported in the lungs of human infants and prenatal mice (6). Given the very low expression of TMPRSS2 in neonatal mice and humans, this may explain why neonates are relatively protected against infection with SARS-CoV-2. Furthermore, our observations support the notion of developmental regulation of TMPRSS2 (6), which may present a mechanistic reason for the observed relative protection of infants and children from the severe form of COVID-19 disease. Moreover, the tightly regulated neonatal immune system (3, 44, 45) may prevent hyper-immune activation and collateral tissue damage associated with COVID-19 pathogenesis (46).

Lastly, our results provide a potential mechanistic explanation for why COVID-19 patients treated with dexamethasone may have different clinical outcomes. It is reported that dexamethasone provides benefits only to COVID-19 patients receiving oxygen therapy and/or invasive mechanical ventilation (23). These patients are in the inflammatory phase of the disease by the time they require respiratory support. This suggests that at this stage COVID-19 disease is dominated by immunopathological alterations and not by active viral replication. Therefore, we speculate that dexamethasone treatment at this phase of the disease may upregulate ACE2 and TMPRSS2 expression, and subsequently counterbalance the increase in RAS activity by converting pro-inflammatory angiotensin II to anti-inflammatory angiotensin (40). Conversely, our data suggest that dexamethasone treatment in the early stage of COVID-19 may be more harmful than helpful. Treatment given at a time when viral entry and replication are high can be detrimental as the upregulation of ACE2 and TMPRSS2 may enhance infection. In support of this concept, slower clearance of virus has been reported in individuals with influenza, SARS, and the Middle East respiratory syndrome (MERS) treated with systemic glucocorticoids (47–50).

Another explanation for the differential effects of dexamethasone on SARS versus SARS-CoV-2 might be related to differential viral replication phases. In contrary to SARS, in which viral replication peaks in the second week of disease (51), viral replication in SARS-CoV-2 is more pronounced early in the disease and falls rapidly thereafter (52–54). Therefore, our results suggest that dexamethasone may be beneficial only at the right time and in the right patient. We, however, caution against extrapolating these results to COVID-19 patients with other underlying conditions and/or patients with other viral respiratory infections.

The observed increase in expression of ACE2 and TMPRSS2 due to dexamethasone suggests that individuals receiving this treatment for other clinical reasons may be more susceptible to SARS-CoV-2 infection, however, this merits further investigation. Dexamethasone is one of the WHO essential medicines and is readily available, however, our results support its use in the later phase of COVID-19 when viral replication is low and inflammation high. However, in addition to its well-defined anti-inflammatory properties (25), our study provides novel evidence that treatment with dexamethasone can upregulate the expression of ACE2 and TMPRSS2 in lung and heart tissues. Therefore, giving dexamethasone at a time when viral replication is high and viral control is essential could be harmful to the patient.

We are aware of multiple study limitations such as the lack of study on human tissues. Although our studies support the upregulation of ACE2 and TMPRSS2, we were unable to examine the precise effects of dexamethasone treatment on ACE2 and TMPRSS2 localization and different immune/non-immune cells since our studies were performed on bulk tissue. Because dexamethasone may exhibit differential effects on ACE2/TMPRSS2 depending on the target cell (e.g. enhancing the maturation of erythroid progenitors) (29, 55, 56). Also, we did not investigate mechanistically how dexamethasone influences the upregulation of ACE2 and TMPRSS2. Thus, continued investigation into the mechanism associated with enhanced upregulation of ACE2 and TMPRSS2 in the lungs and hearts may shed light on a potential therapeutic approach focused on the modulation of ACE2 and TMPRSS2 expression. Another element that should be taken into consideration is the duration of treatment. Although we examined the effects of dexamethasone following two consecutive days of treatment, human subjects normally receive this medication for a longer period of time (the standard course is 10 days), which may exhibit more pronounced effects. It is also important to acknowledge that the administered dexamethasone dose in animals was higher than that prescribed in COVID-19 patients. However, steroids are usually administered intravenously (IV) in COVID patients, resulting in the highest bioavailability and predictable drug levels. Due to the impracticality in neonatal mice, we administered dexamethasone into animals *via* the IP route. It is well documented that the rate of absorption is much lower by IP versus the IV routes. The rate of absorption after IP injection can be 50% to 25% lower compared with IV administration (57, 58). Another factor that should be taken into consideration is how the administered dexamethasone from the peritoneal cavity gets absorbed. It may face one of two pathways to reach the systemic circulation. It is absorbed through the visceral peritoneum, the mesentery and omentum and is drained into the portal circulation, or the compound may bypass the liver if absorbed through the parietal peritoneum and lymphatics (59). As such it is impossible to assess how much of the drug undergoes hepatic metabolism. Perhaps the systemic dexamethasone bioavailability might be comparable in treated animals in our study to patients, or very close. In support of this hypothesis, we observed significant increase in ACE2 and TMPRSS2 expression levels in NHBE cells following a single treatment with lower dexamethasone concentration (1 μg/ml). Mice are not normally susceptible to SARS-CoV-2 infection and we were unable to conduct such studies in other animal models. Such studies are needed to better investigate the role of dexamethasone *in vivo*. Recently, the effects of dexamethasone treatment in a humanized mouse model of SARS-CoV-2 infection was examined (60). The authors concluded that the initiation of dexamethasone at day 3 post-infection when the viral load is still high was associated with moribund but the same treatment starting at day 7 post infection when the viral load had declined was protective (60). Although the impact of dexamethasone treatment on ACE2 and TMPRSS2 was not examined, it suggests that upregulation of ACE2/TMPRSS2 might be a contributing factor to these observations.

It is intriguing to question whether dexamethasone or other glucocorticoids modulate and upregulate the expression of ACE2 in the cilia of the nasal and upper respiratory tract the same as the lung and heart tissues. If this is the case, individuals with other underlying conditions, even children who are on dexamethasone, may be at higher risk of SARS-CoV-2 infection. The higher expression of ACE2 in neonatal hearts and upregulation of ACE2 in the heart of neonatal versus adult mice may suggest a higher affinity of the spike protein for the heart tissues of infants, in particular, when on dexamethasone. However, it’s unclear whether other glucocorticoids exhibit similar effects on ACE2 and TMPRSS2 expression in tissues. Moreover, the expression of ACE2 and TMPRSS2 is not restricted to the lung and heart tissues but other organs (e.g. liver and kidney) and different cells (55, 56). Therefore, examining the broader effects of dexamethasone treatment on the expression levels of ACE2 and TMPRSS2 in immune and non-immune cells merits further investigation.

**Figure 8:**
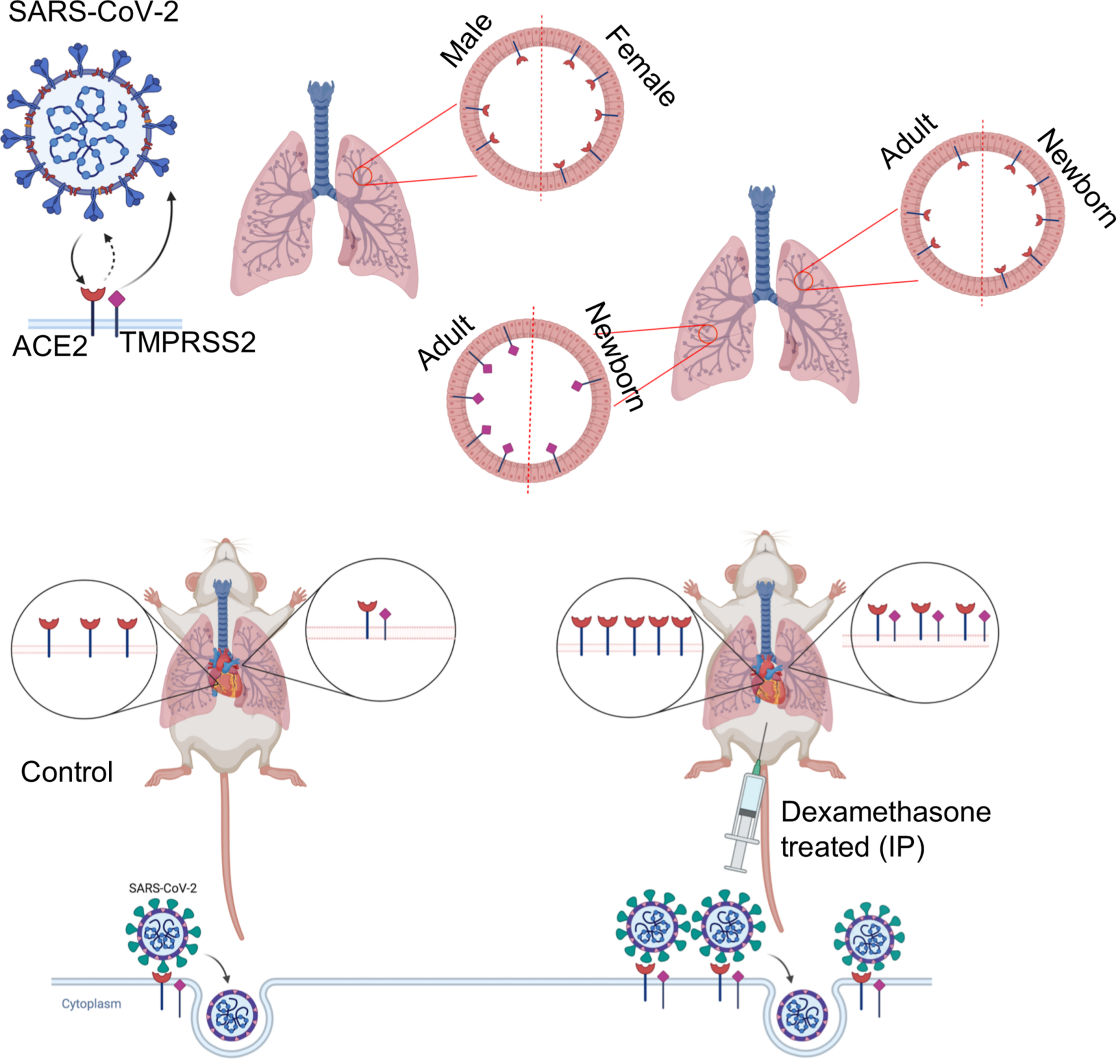
The visual summary of the study.

In conclusion, we found that ACE2 expression in the lung and heart varies in relation to sex and age. The identification of developmental regulation of TMPRSS2 expression could partially explain the differential susceptibility of neonates to SARS-CoV-2 infection. Moreover, we reveal *in vivo* evidence that dexamethasone enhances the expression of ACE2 and TMPRSS2 (**Figure 8**), and subsequently the infectivity of NHBE and Vero E6 cells to pseudo SARS-CoV-2. These observations suggest that this medication might exhibit differential therapeutic effects depending on the phase of COVID-19. Finally, our results suggest that dexamethasone treatment for other clinical purposes may enhance the susceptibility of those patients to SARS-CoV-2 infection.

## Data availability statement

The original contributions presented in the study are included in the article/supplementary material. Further inquiries can be directed to the corresponding author.

## Ethics statement

The animal study was reviewed and approved by the research ethics boards at the University of Alberta that approved these studies with the protocol # AUP0001021.

## Author contributions

ShS performed PCR, flow cytometry related, and *in vitro* infection studies. OO performed western blotting and flow cytometry studies. SS assisted in *in vitro* infection studies. MO and WS provided resources and inputs in experimental design. SE conceptualized the study, designed the study approach, provided resources, supervised the study, designed the figures and wrote the manuscript. All authors contributed to the article and approved the submitted version.

## Funding

This work was supported by the Canadian Institutes of Health Research (CIHR, grant #453061) and a LI Ka Shing Institute of Virology Innovation grant (both to SE).

## Acknowledgments

The authors would like to thank the University of Alberta, Faculty of Medicine and Dentistry’s Flow cytometry facility.

## Conflict of interest

The authors declare that the research was conducted in the absence of any commercial or financial relationships that could be construed as a potential conflict of interest.

## Publisher’s note

All claims expressed in this article are solely those of the authors and do not necessarily represent those of their affiliated organizations, or those of the publisher, the editors and the reviewers. Any product that may be evaluated in this article, or claim that may be made by its manufacturer, is not guaranteed or endorsed by the publisher.
